# Co-morbidity but not dysglycaemia reduces quality of life in patients with type-2 diabetes treated with oral mono- or dual combination therapy – an analysis of the DiaRegis registry

**DOI:** 10.1186/1475-2840-12-47

**Published:** 2013-03-20

**Authors:** Jürgen Wasem, Peter Bramlage, Anselm K Gitt, Christiane Binz, Michael Krekler, Evelin Deeg, Diethelm Tschöpe

**Affiliations:** 1 Lehrstuhl für Medizinmanagement, Universität Duisburg-Essen, Essen, Germany; 2 Institut für Pharmakologie und präventive Medizin, Mahlow, Germany; 3 Institut für Herzinfarktforschung Ludwigshafen an der Universität Heidelberg, Ludwigshafen, Germany; 4 Bristol-Myers Squibb, Medical Department, Munich, Germany; 5 Stiftung „Der herzkranke Diabetiker“in der Deutschen Diabetes Stiftung, Bad Oeynhausen, Germany; 6 Herz- und Diabeteszentrum Nordrhein-Westfalen in Bad Oeynhausen, Universitätsklinik der Ruhr Universität Bochum, Bochum, Germany

## Abstract

**Background:**

Type-2 diabetes mellitus has a major impact on health related quality of life (HRQoL). We aimed to identify patient and treatment related variables having a major impact.

**Methods:**

DiaRegis is a prospective diabetes registry. The EQ-5D was used to describe differences in HRQoL at baseline. Odds ratios (OR) with 95% confidence intervals (CI) were determined from univariable regression analysis. For the identification of independent predictors of a low score on the EQ-5D, multivariable unconditional logistic regression analysis was performed.

**Results:**

A total of 2,760 patients were available for the present analysis (46.7% female, median age 66.2 years). Patients had considerable co-morbidity (18.3% coronary artery disease, 10.6% heart failure, 5.9% PAD and 5.0% stroke/TIA). Baseline HbA1c was 7.4%, fasting- and postprandial plasma glucose 139 mg/dl and 183 mg/dl.

The median EQ-5D was 0.9 (interquartile range [IQR] 0.8–1.0). Independent predictors for a low EQ-5D were age > 66 years (OR 1.49; 95%CI 1.08–2.06), female gender (2.11; 1.55–2.86), hypertension (1.73; 1.03–2.93), peripheral neuropathy (1.62; 0.93–2.84) and clinically relevant depression (11.01; 3.97–30.50). There was no influence of dysglycaemia on the EQ-5D score.

**Conclusion:**

The present study suggests, that co-morbidity but not average glycaemic control reduces health related quality of life in type 2 diabetes mellitus.

## Introduction

In the treatment of type-2 diabetes there is a strong focus to achieve glycaemic control and to prevent directly quantifiable disease related micro- and macrovascular complications. The achievement of these goals is perceived to be an indicator of a good quality of care. However, this reflects disease control rather than a patient’s health related quality of life (HRQoL) [1] and patients’ perceived HRQoL does not necessarily match patients’ ratings [2].

There are a number of studies showing that HRQoL is reduced in type-2 diabetic patients compared to the general population [3] and also somewhat lower than in patients with other chronic disease entities [4]. However, the relationship between glycaemic control and HRQoL of patients with type 2 diabetes is unclear. It has been suggested that reducing glucose to normal levels may enhance well-being, particularly as fatigue is frequent in patients with elevated blood glucose levels [5]. But while some studies have shown that improved HbA1c is associated with short-term improvement in HRQoL [6], others were not able to confirm this [7]. Further antidiabetic treatment such as sulfonylureas might impact HRQoL because of a potentially increased body weight and the risk of hypoglycaemia [8,9]. More recent drug developments such as DDP-4 inhibitors on the other hand rather decrease body weight and patients have a low propensity for hypoglycaemia which might have a positive impact on HRQoL. The most consistent impact on HRQoL has been reported for co-morbidities [10-13] and disease-related complications [14]. In multiple regression analyses for example, microvascular complications, heart failure and depression were most strongly related to decreased HRQoL, independently of the duration of diabetes while patients without symptomatic co-morbidities did not have a significantly reduced HRQoL [10]. In agreement with this, symptomatic co-morbidities such as osteoarthritis and neuropathic pain have a stronger impact on HRQoL than largely asymptomatic co-morbidities such as hypertension [12].

Given the vast but partially conflicting evidence on HRQoL from a number of different cohorts and research settings we used the DiaRegis dataset consisting of a large cohort of type-2 diabetic patients on oral antidiabetic pharmacotherapy [15] to identify independent patient and treatment related variables that affect HRQoL.

## Methods

DiaRegis is a prospective, observational, German, multicenter registry. It is conducted in accordance with the principles of Good Epidemiology Practice (GEP), and applicable regulatory requirements. The protocol of this registry was approved by the ethics committee of the Landesärztekammer Thüringen in Jena, Germany on March 4th 2009 [15,16]. Patients being enrolled into this registry provided written informed consent.

### Patients

Between June 2009 and March 2010 patients with type-2 diabetes aged ≥ 40 years being on oral mono or dual combination antidiabetic therapy (no injectables such as insulin or GLP-1 analogs) were included in which the treating physician intensified treatment at baseline. Intensification was achieved by either increasing the dose of originally prescribed drugs and/or by exchanging drugs or by adding further drugs to the previously used ones. There was no interaction with the physician to select particular patients to be intensified, nor the direction of intensification pre-defined. All eligible patients were enrolled on a consecutive basis. Patients not under regular supervision of the treating physician for the duration of the study, those with type-1 diabetes, pregnancy, diabetes secondary to malnutrition, infection or surgery, with maturity onset diabetes of the young, known cancer or limited life expectancy, acute emergencies, participation in a clinical trial and patients with further reasons that made it impossible or highly problematic for the patient to participate and come to the follow-up visits were excluded.

### Documentation

Patient characteristics at baseline were entered via a secure website directly into an electronic database at the *Stiftung für Herzinfarktforschung*, Ludwigshafen, Germany. At this stage they were automatically checked for plausibility and completeness. Data from the patient questionnaire (paper version), which was asked to be completed by the patient during the visit, were transferred to the responsible CRO. The questionnaires were scanned, decoded and transferred to the *Institut für Herzinfarktforschung* for evaluation.

### The EQ-5D (EuroQol)

The EQ-5D [17,18] was used to evaluate health related quality of life (HR-QoL) in an analysis of the DiaRegis baseline population. The EQ-5D is one of the world widest used standardised, validated generic instruments for measuring HRQoL. It consists of a descriptive system (part I) and a visual analogue scale (VAS) (part II). The descriptive system comprises 5 dimensions (mobility, self-care, usual activities, pain / discomfort, anxiety / depression), each being classified as no problems (score 1), some problems (score 2) or severe problems (score 3). The EQ VAS records the respondent´s self rated health on a vertical, visual analogue scale, where the endpoints are labelled “Best imaginable” and “Worst imaginable” health status. The EQ-5D score ranges from 0 to 1 and can be calculated by applying scores from the EQ-5D preference weights elicited from the general population. For this study, the EQ-5D score was calculated using the value set for the European population [17,18]. Further investigations have demonstrated the usefulness of the EQ-5D in identifying determinants of health states [19,20]. The minimal important difference for the EQ-5D has been reported in the relevant literature as a change in score of at least 0.05 points [21].

### Statistical analysis

The statistical analysis was performed using SAS, version 9.2 (Cary, North Carolina, U.S.A.). All descriptive statistics are based on available cases. The distribution of metric variables is described with medians and quartiles. The EQ-5D patient data were analysed using the full model of the validated instrument for the German population [22]. Three tertiles were built to create patient groups with worse, average and above average QoL.Cochran-Armitage or Jonckheere-Terpstra tests were used to describe differences across these tertiles. Odds ratios (OR) with 95% confidence intervals were determined from univariable regression analysis. For risk analyses with HRQoL as outcome variable, multivariable unconditional logistic regression analysis was performed. Variables were selected from univariable analysis and amended by variables derived from clinical considerations. To facilitate interpretation and presentation of data, continuous variables were categorized according to clinically relevant classes.

## Results

Out of 3,810 patients overall, 2,760 patients had complete data for the present analysis of which 1,288 (46.7%) were female (Table 1). Median age was 66.2 (interquartile range [IQR] 57.6–75.6) years and the BMI 30.0 (27.0–35.0) kg/m^2^. The median HRQoL as determined by the EQ-5D was 0.9 (IQR 0.8–1.0).

**Table 1:** Patient characteristics and variables predictive for a reduced EQ-5D score based on univariable and multivariable unconditional regression analysis

	**Total population**	**Regression analysis for having a low score on the EQ-5D**		
	**Median (quartiles) or %**	**Comparison**	**Univariable**	**Multivariable**
			**OR (95%CI)**	**OR (95%CI)**
Age (years)	66.2 (57.6–73.0)	> 66 vs. ≤ 66 years	1.77 (1.47–2.13)	1.49 (1.08–2.06)
Female gender	46.7	female vs. male	1.86 (1.86–2.24)	2.11 (1.55–2.86)
BMI > 30 kg/m^2^	49.3	> 30 vs. ≤ 30 kg/m^2^	1.40 (1.16–1.68)	1.24 (0.91–1.69)
Blood glucose				
HbA1c (%)	7.4 (6.8–8.2)	> 7.4 vs. ≤ 7.4%	0.97 (0.80.1.16)	0.91 (0.64–1.28)
Fasting plasma glucose (mg/dl)	139 (118–167)	> 139 vs. ≤ 139 mg/dl	1.05 (0.86–1.28)	0.91 (0.64–1.29)
Postprandial plasma glucose (mg/dl)	183 (154–220)	> 183 vs. ≤ 183 mg/dl	1.07 (0.84–1.37)	1.20 (0.85–1.71)
Antidiabetic Pharmacotherapy				
Metformin	84.6	yes vs. no	0.81 (0.63–1.61)	0.94 (0.57–1.56)
Sulfonylureas	28.1	yes vs. no	1.13 (0.92–1.39)	1.14 (0.75–1.71)
Glucosidase inhibitors	2.2	yes vs. no	1.19 (0.61–2.29)	1.71 (0.56–5.18)
Glinides	4.0	yes vs. no	1.02 (0.63–1.66)	1.12 (0.50–2.52)
Thiazolidinediones	6.4	yes vs. no	0.93 (0.64–1.35)	1.60 (0.90–2.84)
DPP-4 inhibitors	5.0	yes vs. no	1.11 (0.73–1.7)	0.97 (0.46–2.04)
Co-morbidities				
Hypertension	85.0	yes vs. no	1.83 (1.40–2.40)	1.62 (0.93–2.84)
CAD	18.3	yes vs. no	2.21 (1.73–2.80)	1.39 (0.83–2.31)
Peripheral neuropathy	13.7	yes vs. no	2.51 (1.91–3.3)	1.73 (1.03–2.93)
Heart failure	10.6	yes vs. no	2.01 (1.50–2.70)	1.14 (0.67–1.95)
PAD	5.9	yes vs. no	1.89 (1.28–2.82)	1.46 (0.76–2.81)
Clin. rel. depression	5.3	yes vs. no	10.16 (5.38–19.18)	11.01 (3.97–30.50)
Stroke/TIA	5.0	yes vs. no	1.98 (1.29–3.04)	1.37 (0.69–2.73)
Non-proliferative retinopathy	3.9	yes vs. no	1.76 (1.09–2.83)	1.08 (0.54–2.15)
Autonomous neuropathy	3.4	yes vs. no	2.72 (1.57–4.72)	1.34 (0.56–3.17)
Limb amputation	0.8	yes vs. no	2.38 (0.81–6.99)	0.64 (0.13–3.27)
≥ 2 co-morbidities*	68.2	yes vs. no	1.90 (1.55–2.33)	1.33 (0.77–2.29)
CV Pharmacotherapy				
ARB	22.3	yes vs. no	1.16 (0.93–1.45)	1.20 (0.78–1.87)
ACE inhibitors	50.3	yes vs. no	1.34 (1.11–1.61)	1.08 (0.74–1.58)
Beta-Blocker	47.0	yes vs. no	1.58 (1.31–1.90)	0.88 (0.64–1.23)
CCB	26.2	yes vs. no	1.52 (1.23–1.88)	1.29 (0.92–1.81)
Diuretics	41.2	yes vs. no	1.60 (1.32–1.93)	1.09 (0.78–1.52)
Statins	41.7	yes vs. no	1.28 (1.06–1.54)	1.06 (0.77–1.46)
Anamn. Hypoglycaemia	11.7	yes vs. no	2.01 (1.51–2.66)	1.40 (0.88–2.22)

*Legend:* *co-morbidities considered are listed under “co-morbidities” in the table; *IQR* interquartile range; *OR* odds ratio; *CI* confidence interval; *BMI* body mass index; *DPP-4* dipeptidyl-peptidase 4 inhibitor; *CAD* coronary artery disease; *PAD* peripheral arterial disease; *TIA* transitory ischemic attack; *CV* cardiovascular; *ARB* angiotensin receptor blocker; *CCB* calcium channel blocker.

### Impact of glycaemic control

Median HbA1c was 7.4 (IQR 6.8–8.2) in the total cohort, which was not different between tertiles of the EQ-5D (p = 0.97). Likewise no relationship between fasting- (p = 0.80) or postprandial plasma glucose (p = 0.16) and EQ-5D was observed (Table 1). In multivariable regression analyses considering variables depicted in Table 1, the OR was 0.91 (0.64–1.28) for the HbA1c, 0.91 (0.64–1.29) for fasting- and 1.20 (0.85–1.71) for postprandial plasma glucose indicating no considerable impact.

### Impact of hypoglycemia

**Figure 1:**
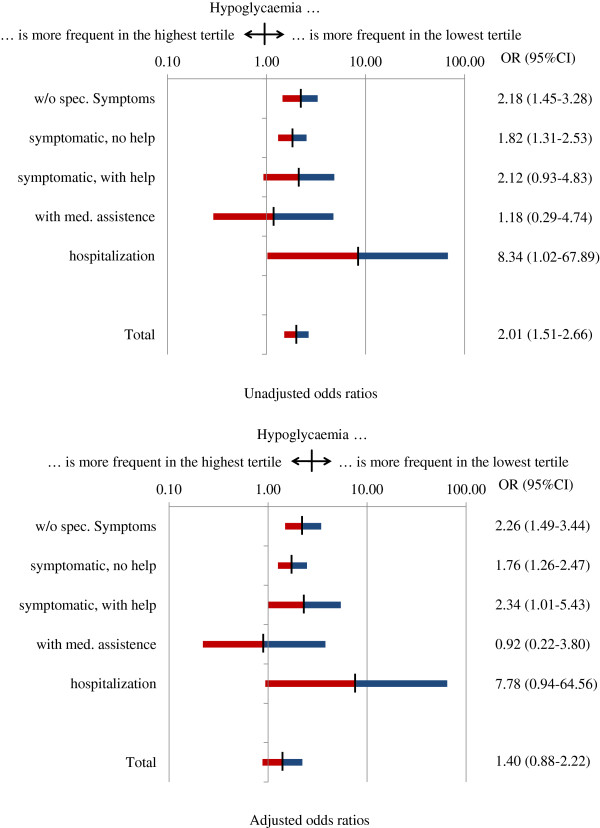
**HRQoL in patients with hypoglycaemia.** Legend: Odds Ratios in the lower panel were adjusted for an age, gender and body mass index.

Physicians reported that 11.7% of patients had suffered from episodes of hypoglycaemia during the 12 months prior to inclusion, which were more frequent in patients with an EQ-5D score in the lowest tertile (16.7% vs. 9.1% in the highest tertile; OR 2.01; 95%CI 1.51–2.66) (Figure 1). After adjusting for age, gender and body mass index the association with asymptomatic hypoglycaemia (OR 2.26; 95%CI 1.49–3.44) and symptomatic hypoglycaemia without the need for help (1.76; 1.26–2.47) remained significant, while symptomatic hypoglycaemia with the need for help became significant (2.34; 1.01–5.43). A need for hospitalization had a high OR assigned but confidence intervals were wide due to low patient numbers. In the multivariable model depicted in Table 1 hypoglycaemia lost its association with a reduced EQ-5D score.

### Impact of antidiabetic pharmacotherapy

84.6% of patients received metformin, 28.1% sulfonylureas, 2.2% glucosidase inhibitors, 4.0% glinides, 6.4% glitazones and 5.0% DPP-4 inhibitors (Table 1). Neither of these compounds was associated with a reduced EQ-5D score, which was confirmed in univariable and also multivariable regression analyses. This also applied for 69.7% of patients receiving oral monotherapy and 30.3% receiving any oral combination therapy.

### Impact of co-morbidity and patient related variables on the EQ-5D score

**Figure 2:**
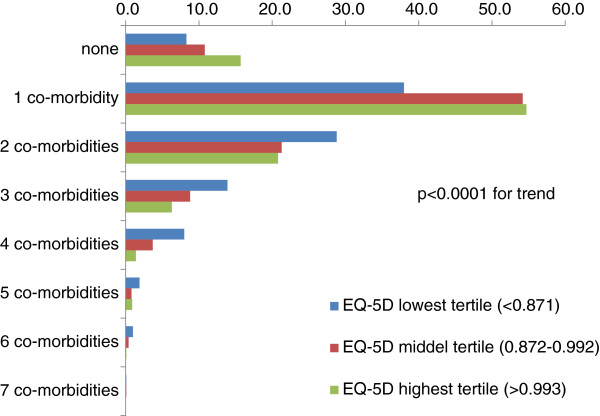
**Co-morbidity burden and health related quality of life (EQ-5D).***Legend:* co-morbidities considered were dyslipidemia, hypertension, malignancy, coronary artery disease, stroke/transitory ischemic attack (TIA), peripheral artery disease (PAD), heart failure, neuropathy, retinopathy, dialysis, clinically relevant depression.

Patients had a considerable co-morbidity burden (Figure 2), which was higher in patients with a low score on the EQ-5D. Patients with a reduced score were older, more often female and had a higher median BMI and had coronary artery disease, stroke/transitory ischemic attack (TIA), peripheral artery disease (PAD), heart failure and peripheral neuropathy more frequent (Table 1). After multivariable analysis hypertension (OR 1.62; 95%CI 0.93–2.84), peripheral neuropathy (OR 1.73, 95%CI 1.03–2.93) and clinically relevant depression (OR 11.01; 95%CI 3.97–30.50) but not heart failure or coronary artery disease (CAD) remained significant predictors. Table 2 lists the items of the EQ-5D per co-morbidity status and reveals a substantially altered response pattern which was present troughout all domains, but particularly pronounced in patients with pain/discomfort.

**Table 2:** EQ-5D items in patients per co-morbidity status

	**Total**	**0 or 1 co-morbidity**	**2 co-morbidities**	**> 2 co-morbidities**	**p-value**
	**(n = 2760)**		**(n = 646)**	**(n = 424)**	
		**(n = 1690)**			
Mobility					
I have no problems in walking about	72.3	79.5	66.9	52.1	<0.0001
I have some problems in walking about	27.6	20.4	33.1	47.6	<0.0001
I am confined to bed	0.1	0.1	0.0	0.2	0.77
Self-Care					
I have no problems with self-care	92.2	94.9	91.2	83.0	<0.0001
I have some problems washing or dressing myself	7.4	4.7	8.7	16.0	<0.0001
I am unable to wash or dress myself	0.5	0.5	0.2	0.9	0.46
Usual Activities					
I have no problems performing my usual activities	80.8	86.7	75.7	64.9	<0.0001
I have some problems performing my usual activities	17.9	12.3	23.7	31.4	<0.0001
I am unable to perform my usual activities	1.3	0.9	0.6	3.8	<0.001
Pain/Discomfort					
I have no pain or discomfort	43.3	48.4	39.5	28.8	<0.0001
I have moderate pain or discomfort	50.1	46.3	52.5	61.8	<0.0001
I have extreme pain or discomfort	6.6	5.3	8.0	9.4	<0.001
Anxiety/Depression					
I am not anxious or depressed	72.6	76.1	68.9	64.6	<0.0001
I am moderately anxious or depressed	24.8	22.0	27.1	32.5	<0.0001
I am extremely anxious or depressed	2.6	2.0	4.0	2.8	0.06
EuroQoL EQ-5D visual analogue scale	70 (55–80)	73 (60–80)	70 (53–80)	60 (50–70)	<0.0001
EQ-5D Score (Full Model) [0–1]	0.9 (0.8–1.0)	0.9 (0.9–1.0)	0.9 (0.8–1.0)	0.9 (0.8–0.9)	<0.0001

Concomittant cardiovascular pharmacotherapy such as ACE inhibitors, betablockers, calciumchannel blockers, diuretics and statins were more frequent in patients with a low EQ-5D score. They were however rather reflecting the substantial co-morbidity of patients because all associations became insignificant after multivariable analysis.

## Discussion

Type-2 diabetes has been described to be associated with a reduced health related quality of life [3,4]. Using the EQ-5D questionnaire we found that this was mostly related to the presence of co-morbidity and episodes of hypoglycaemia but not to average glycaemic control. With a median score of 0.9 diabetic patients scored high on the EQ-5D in general while there were only few patients with substantially lowered scores (<0.5). On the other hand changes as little as 0.05 have been reported as the minimal important difference for the EQ-5D [21], illustrating that there are clinically relevant differences in HRQoL between those in the lowest and highest tertile of the EQ-5D range seen in DiaRegis.

### Glycaemic control

We found no association between glycaemic control (defined as HbA1c, fasting- or postprandial glucose levels) and changes in the EQ-5D scoring, which remained robust even in multivariable analyses. While the findings are in agreement with a number of other analyses [4,23,24], they are in contrast to data reported by Testa et al., who found that improved glycaemic control was associated with substantial improvements in QoL [6]. Quality-of-life treatment differences (SD units) for symptom distress (+0.59; p < 0.001), general perceived health (+0.36; p = 0.004), cognitive functioning (+0.34; p = 0.005), and the overall visual analog scale (VAS) (+0.24; p = 0.04) were significantly more favorable for active therapy. In that study patients with established type-2 diabetes were washed out from prior antidiabetic pharmacotherapy for 3 weeks and then randomized to glipizide GITS or placebo for 12 weeks. HbA1c increased to 9.3% and fasting blood glucose to 168 mg/dl in the placebo group while it was reduced to 7.5% and 126 mg/dl in the glipizide group. In another analysis by Klein et al. diabetic patients after 14 years of follow-up were subjected to the Short Form 36 (SF-36), which demonstrated an improved quality of life with low HbA1c values [25], this was however not multivariable adjusted.

On this background the lack of an association between glycaemic control and HRQoL in our study might be related to the fact that the group of patients was rather homogenous, selected by restricting recruitment to those with oral antidiabetic therapy, and showing a rather narrow range of HbA1c values (median 7.4; interquartile range 6.8–8.2) that appeared to be stable over time. Taken together this might suggest that average contemporary glycaemic control in a stable environment might prevent detecting differences in QoL that may arise in patients with extreme differences in glycaemic control.

### Antidiabetic pharmacotherapy

Differences in HRQol with the selection of oral antidiabetic pharmacotherapy are, on the background of a largely absent impact of glycaemic control, related to the side effect profile which might include weight gain and/or hypoglycaemia. In the analysis of our dataset which included patients being largely treated with metformin (84.6%) and/or sulfonylureas (28.1%) and also to a lesser extent with other oral antidiabetic drugs, we found no impact of antidiabetic pharmacotherapy in univariable or multivariable regression analyses. This is compatible with other analyses of observational registries and larger outcomes studies which also identified no such association [4,26,27].

Hypoglycemia is a frequent treatment related complications that might be reduced by properly selecting antidiabetic pharmacotherapy and considering specific patient characteristics use [28,29]. There are only few reports demonstrating that hypoglycaemia with oral antidiabetic drugs might have an impact. Alvarez-Guisasola et al. reported for example from a large observational, multicenter, cross sectional study including 1,709 patients with type-2 diabetes (mean age 63 years, 45% female, mean HbA1c 7.1%, 38% hypoglycaemic symptoms within the last 12 months), that QoL was substantially reduced with hypoglycaemia [30]. This is well compatible with our own observations. Patients received either sulfonylureas or thiazolidinediones on top of metformin monotherapy. Those reporting hypoglycaemic symptoms had significantly lower EQ-5D VAS scores (mean difference -4.33, p < 0.0001) in adjusted linear regression analyses. Relative to those not reporting symptoms, the adjusted decrement to quality of life increased with severity of hypoglycaemia (mild: -2.68, p = 0.0039; moderate: -6.42, p < 0.0001; severe: -16.09, p < 0.0001). They established no link however between the choice of either sulfonylureas or glitazones and the frequency of hypoglycaemia. Further research is clearly needed to link antidiabetic treatment options to hypoglycaemia and their impact on health related quality of life. It is however plausible that the incidence rates of (symptomatic) hypoglycaemia with single agents in our cohort is not sufficient to establish such a link from a statistical perspective.

### Co-morbidity and patient related variables

Patients in our cohort had a substantial co-morbidity which ranged however from none to more than 6 co-morbidities. After multivariable analysis hypertension (OR 1.62; 95%CI 0.93–2.84), peripheral neuropathy (OR 1.73, 95%CI 1.03–2.93) and clinically relevant depression (OR 11.01; 95%CI 3.97–30.50) but not heart failure or coronary artery disease (CAD) remained significant predictors. This is in partial agreement to previous findings [10-14] that have repeatedly shown, that symptomatic more than asymptomatic co-morbidities are responsible for this observation. Miksch for example illustrated that the impact of osteoarthritis as a disabling and painful condition reduces QoL more than hypertension which is largely asymptomatic [12]. Wexler et al. also found that patients with symptomatic co-morbidities such as microvascular complications and heart failure had a substantially reduced QoL, while those without symptoms showed no reduction [10]. These findings are also consistent with our observation that peripheral neuropathy had a higher impact on HRQoL than coronary artery disease and even heart failure. It was surprising however for us to find that heart failure did not reduce quality of life in our multivariable model while it did in the univariable analyses, because it is in contrast to previous reports [10]. This might have been due to multiple adjustments in the multivariable model, which reduced the impact of heart failure.

We were not able to show a direct impact of physical activity on HRQoL, which was elegantly demonstrate by Daniele et al. [31], who found an improved HRQoL in physically active patients with diabetes. This might provide, beyond improvements of metabolic control and body weight, an opportunity to improve HRQoL.

## Conclusions

The present study suggests, that co-morbidity but not average glycaemic control reduces health related quality of life in type 2 diabetes mellitus.

## Competing interests

Jürgen Wasem, Peter Bramlage, Anselm K. Gitt and Diethelm Tschöpe have received research support and honoraria for lectures from a number of pharmaceutical companies including Bristol-Myers Squibb and AstraZeneca, the sponsors of the present registry. Christiane Binz and Michael Krekler are employees of the sponsors. Evelin Deeg has no potential conflict of interest to disclose.

## Authors’ contributions

PB, AKG, DT, CB, and MK have been deeply involved in the conception and design of the study. ED was responsible for the analysis of data. JW and PB have drafted the manuscript and all other authors have been revising the article for important intellectual content. All authors read and approved the final manuscript.
